# Auditory hallucinations in a young person with Jacobsen Syndrome and Partial Trisomy 10q Syndrome

**DOI:** 10.1192/j.eurpsy.2025.1175

**Published:** 2025-08-26

**Authors:** J. Sánchez-Cerezo, P. Del Sol Calderon

**Affiliations:** 1 Division of Child and Adolescent Psychiatry, Department of Psychiatry, Hospital Universitario Puerta de Hierro, Majadahonda, Madrid, Spain

## Abstract

**Introduction:**

Jacobsen syndrome (JS) is a rare genetic disorder caused by a partial deletion of the long arm of chromosome 11 (11q23.3qter), affecting approximately 1 in 100,000 births. It is associated with physical and developmental abnormalities, including pre- and postnatal growth retardation, facial dysmorphism, and multiple congenital malformations. Intellectual disability and psychomotor retardation are also common, with 97% of individuals presenting with varying degrees of cognitive impairment. Autism spectrum disorder (ASD), attention deficit hyperactivity disorder (ADHD), and, in rare cases, severe psychiatric disorders like schizophrenia or bipolar disorder, may complicate the clinical picture. JS management is complex, often requiring multidisciplinary care. Partial trisomy 10q is a rare chromosomal disorder, with around 40 reported cases worldwide. It is characterized by distinctive facial features, minor physical anomalies, and possible cardiac or renal malformations, with severity depending on the duplicated region.

**Objectives:**

The aim of this case report is to present a 13-year-old female with Jacobsen syndrome and Partial Trisomy 10q Syndrome who was diagnosed with ASD and intellectual disability (ID) who later developed psychotic symptoms.

**Methods:**

The patient, a 13-year-old girl, was diagnosed with Jacobsen syndrome and Partial Trisomy 10q Syndrome following a neurological evaluation conducted at two months of age. Medical records were reviewed from her initial assessments to recent follow-ups.

**Results:**

The patient has ASD and ID, with poor verbal communication, repetitive behaviours, and social isolation. A notable regression in both language and behaviour was observed after the age of 10, coinciding with her school integration. Neuroimaging showed ventriculomegaly and loss of white matter, but no active demyelination or epileptic features were found. Due to impaired attention, we suspected ADHD, and started her on methylphenidate, later changing it to atomoxetine with poor response. Due to patient’s very limited speech, mental state examination was difficult. We observed that she was anxious, had hearing phenomena, and was talking in jargon, so we suspected psychotic symptoms in the form of auditory hallucinations. Treatment with Aripiprazole was initiated at doses of up to 7 mg daily, with a good response. Aripiprazole also helped to reduce social withdrawal and improve attention. Currently, the patient is maintained on a stable dose of Aripiprazole, alongside behavioural therapies and educational support.

**Conclusions:**

This case highlights the complexity of managing young people with neuropsychiatric symptoms in patients with genetic syndromes, especially when poor language and speech skills are associated. Future research is needed to better understand the neuropsychiatric implications of JS Partial Trisomy 10q Syndrome and optimize treatment strategies for these patients.

**Disclosure of Interest:**

None Declared

